# Computational modeling of cough-induced droplets and mucosal film dynamics in the upper airway for pulmonary disease classification

**DOI:** 10.3389/fphys.2025.1666826

**Published:** 2025-09-19

**Authors:** Olusegun J. Ilegbusi, Rafid Jahangir Khan, Bari Hoffman

**Affiliations:** ^1^ Department of Mechanical and Aerospace Engineering, University of Central Florida, Orlando, FL, United States; ^2^ School of Communication Sciences and Disorders, University of Central Florida, Orlando, FL, United States; ^3^ Department of Internal Medicine, University of Central Florida, Orlando, FL, United States

**Keywords:** cough, cough droplets, mucus, respiratory disease, computational fluid dynamics, non-invasive diagnostics

## Abstract

**Introduction:**

Cough-generated droplets are critical in the transmission and progression of respiratory diseases. This study investigates droplet formation and transport in the upper airway during a cough to improve understanding of their biomechanical behavior and explore their potential for non-invasive classification of airway diseases.

**Methods:**

A computational fluid dynamics model is employed to simulate a transient, droplet-laden cough in a CT-derived human upper airway, using an experimentally acquired cough profile. The method incorporates mucus film dynamics using the Eulerian Wall Film (EWF) model and droplet transport using the Discrete Phase Model (DPM). Three mucus thicknesses—healthy baseline (Type I), intermediate pathological thickening (Type II), and advanced pathological thickening (Type III)—and three viscosity levels for Type II: baseline viscosity (Type II-A), intermediate viscosity (Type II-B), and high viscosity (Type II-C) are considered. These cases represent a progressive increase in both mucus thickness and viscosity, encompassing a spectrum of respiratory conditions.

**Results:**

The results show that a 50% increase in mucus thickness (from 20 μm to 30 μm) results in 4.3-fold increase in exhaled droplet count and a 20% increase in mean droplet size. Conversely, a 50% increase in mucus viscosity reduces exhaled droplet count by 2.7-fold while increasing mean droplet size by 9%. Absorbed droplets, which remain within the airway, exhibit similar trends; however, as they are not measurable non-invasively, their diagnostic utility is limited.

**Discussion:**

These findings highlight the role of mucus in droplet dynamics, with increased thickness and viscosity driving larger droplet sizes, and support the potential of exhaled droplet size distribution as a diagnostic biomarker for airway disease.

## 1 Introduction

Cough is a vital airway protective function that serves to clear mucus, pathogens, and inhaled particulates from the respiratory tract. It is characterized by a rapid sequence of deep inhalation, glottal closure, and forceful exhalation, resulting in high-velocity airflow that facilitates the expulsion of airway debris (Andrani et al., 2019; Chang, 2006; Hegland et al., 2013; Poliacek et al., 2016). This process works in concert with mucociliary clearance, which is driven by the coordinated action of cilia that propels mucus toward the oropharynx (Bustamante-Marin and Ostrowski, 2017; Button and Button, 2013). When mucociliary function becomes compromised, cough becomes the primary mechanism for airway clearance. Together, cough and mucociliary clearance act synergistically to maintain pulmonary hygiene and protect against airway compromise (Sapienza and Hoffman, 2020).

Mucus serves a critical role in respiratory defense by creating a protective barrier, trapping inhaled pathogens and particulates, maintaining airway hydration and facilitating mucociliary transport for clearance (Evans and Koo, 2009; Pangeni et al., 2023; Zanin et al., 2016). In conditions such as chronic obstructive pulmonary disease (COPD), chronic bronchitis, and cystic fibrosis (CF), altered mucus properties, including increased thickness and viscosity due to higher mucin concentration or inflammation, impair clearance mechanisms (Abrami et al., 2024; Batson et al., 2022; Fahy and Dickey, 2010; Ghanem et al., 2021; Hill et al., 2022; Lin et al., 2020; Shah et al., 2023; Tufail et al., 2025). Under healthy conditions, the mucus layer measures approximately 10–20 μm in thickness, but in disease states, it can thicken significantly (Atanasova and Reznikov, 2019; Duncan et al., 2016; Michael Foster, 2002). This thickened mucus promotes pathogen retention and chronic infections while influencing the aerodynamic properties of cough. Changes in mucus viscosity and surface tension can alter the droplet size distribution during cough, with potential implications for disease transmission and respiratory function (Hill et al., 2018; Thomson et al., 2013). This study focuses on the effect of mucus thickness and viscosity on droplet dynamics as a foundational step toward pulmonary disease classification.

During a cough, rapid airflow exerts a shearing force on the mucus layer, creating instabilities that cause the mucus to break into droplets. These instabilities, including Kelvin–Helmholtz instabilities driven by airflow shearing, lead to surface wave fragmentation and droplet formation, influenced by mucus viscosity and thickness (Reyes et al., 2021; Saha et al., 2024). The viscoelastic properties of mucus produce thinner sheets and smaller droplets, stabilizing fragmentation and increasing airborne transmission potential (Kant et al., 2023; Li et al., 2025). The droplets vary widely in size: smaller droplets (<10 µm) evaporate quickly, forming aerosols capable of long-range airborne transmission, while larger droplets (>100 µm) settle rapidly but carry a higher pathogen load, facilitating short-range transmission (Cortellessa et al., 2021; Katre et al., 2021; Li et al., 2021). These droplets, originating primarily from bronchiolar and laryngeal regions, serve as critical vectors for respiratory pathogens, including SARS-CoV-2 (Pöhlker et al., 2023). This study proposes that disease-induced mucus thickening and increased viscosity alters droplet size distribution, offering a potential non-invasive biomarker for airway pathology, an area that is underexplored in prior research.

The dynamics of cough-generated droplets include both exhalation into the external environment and redeposition (absorption) into the airway mucus layer. While exhaled droplets are directly measurable and hold promise as a non-invasive biomarker for airway pathology, absorbed droplets—those that reattach to the mucus layer—may influence airway clearance and pathogen retention within the respiratory tract. This study examines both exhaled and absorbed droplets to provide a comprehensive understanding of droplet dynamics, with a primary focus on exhaled droplet size distribution as a potential diagnostic tool for classifying airway diseases.

Despite their clinical and epidemiological relevance, the biomechanical processes underlying droplet dynamics in the airway remain poorly characterized, particularly in disease states. Experimental studies on droplet generation during expiratory activities, such as those by (Chao et al., 2009; Duguid, 1946; Fairchild and Stampfer, 1987; Loudon and Roberts, 1967; Papineni and Rosenthal, 1997), often yield inconsistent findings due to limitations in direct sampling at the mouth or nose. Measurements are affected by evaporation, dilution, and sampling losses, leading to inaccurate original size distributions. Variations in instruments, protocols, and ambient conditions further contribute to these discrepancies (Xiao et al., 2025). These limitations underscore the need for computational approaches to accurately model the complex interactions between fluid dynamics and mucus properties in anatomically accurate airways.

Computational Fluid Dynamics (CFD) models provide a valuable tool for simulating air–mucus interactions and cough-induced droplet formation and transport dynamics within anatomically realistic airways. Prior CFD studies have modeled airflow and droplet dynamics during a cough (Anzai et al., 2022; Ilegbusi et al., 2021; Jing et al., 2023; Khoa et al., 2023; Kou et al., 2018). The present study uses a CFD-based framework to simulate cough-induced droplet generation and transport in a CT-derived human upper airway model, investigating the effect of disease-induced mucus thickening and increased viscosity on droplet size distribution and dynamics. It aims to elucidate mucus-driven biomechanical changes to enhance pathogen transmission assessments and support the use of exhaled droplet size distribution as a potential, non-invasive diagnostic tool for detecting and classifying airway disease.

## 2 Materials and methods

### 2.1 Airway geometry and mesh

A three-dimensional (3D) model of the human respiratory tract was used to simulate droplet formation and transport during a cough. The original geometry was obtained from a high-resolution anatomical model developed by the U.S. Environmental Protection Agency’s (EPA) Office of Research and Development (ORD). Derived from human medical imaging data, this model extends from the external nares to the alveolar region and captures anatomical variability. The surface mesh provides detailed representations of the nasal and oral cavities, pharynx, larynx, trachea, and three primary airway paths leading to each of the five pulmonary lobes, with branching structures extending to an average of 23 generations.

**FIGURE 1:**
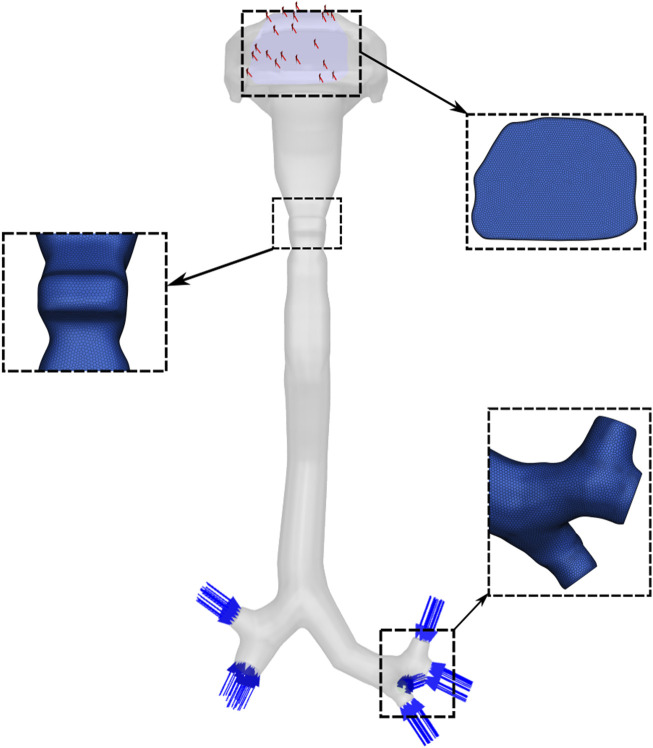
Computational model of the upper airway geometry and mesh.

For the present study, the geometry was reconstructed and modified using Ansys SpaceClaim (ANSYS Inc., Canonsburg, PA, United States). A watertight solid model was created, extending from the oral opening to the third-generation bronchial branches and including seven segmental bronchi. The distal ends of these bronchi were defined as inlets, and the oral cavity opening was specified as the outlet boundary (Figure 1).

An unstructured polyhedral mesh was generated using Ansys Fluent Meshing. A mesh independence study was performed by progressively refining the mesh and comparing velocity fields across key airway cross-sections. The results indicate that a mesh consisting of 2.5 million cells was sufficient for numerical accuracy and was used for all the results presented in the paper.

### 2.2 Mathematical models

The cough flow in the airway is modeled using the continuity (Equation 1) and Navier-Stokes equations (Equation 2). The continuity equation ensures mass conservation, while the Navier-Stokes equations describe the momentum transfer due to viscosity, inertia, and external forces. Given that the temperature change during a cough is relatively small, the energy equation was neglected, assuming that thermal effects on the flow are insignificant. Additionally, the flow was assumed to be incompressible, which is reasonable due to the relatively low Mach number of cough flow. Thus, the governing equations are:

$$\nabla \cdot \vec{v}=0$$ (1)

where $\vec{v}$ is the velocity vector of the fluid.

$$\rho \;\frac{\partial \vec{v}}{\partial t}+\rho \left(\vec{v\;}.\nabla \right)\vec{v\;}=-\nabla p+\nabla .\left(\overset{═}{\tau }\right)+\rho \vec{g}+\vec{F}$$ (2)

where p represents the static pressure, $\overset{═}{\tau }$ denotes the stress tensor, $\rho \vec{g}$ corresponds to the gravitational body force and $\vec{F}$ accounts for the external body forces.

The Eulerian Wall Film (EWF) model was employed to simulate the mucus layer in the airway. The EWF model is designed to simulate the dynamics of liquid films, including their formation, spread, evaporation, and interaction with surrounding airflow. The flow of the liquid (mucus) film is considered two-dimensional along wall surfaces. The film thickness is assumed to be small compared to other geometric dimensions, simplifying the continuity and momentum equations for the film, given as Equations 3, 4, respectively. The film flow accounts for inertia, viscous effects, and gravity. The model predicts film breakup into droplets and their subsequent stripping into the surrounding airflow. The following continuity and momentum equations govern the mucus film dynamics:

$$\frac{\partial h}{\partial t}+\nabla_{s\;.\;}\left[h\;V_{m}\right]=\frac{m_{s}}{\rho_{m}}$$ (3)

where *h* denotes the film height, $V_{m}$ is the average velocity of the mucus film, $\rho_{m}$ is the mucus film density, $m_{s}$ represents the mass source per unit area of the wall, and ∇_S_ signifies the surface gradient operator.

The momentum equation for the mucus film can be written as:

$$\frac{\partial hV_{m}}{\partial t}+\nabla_{s\;.\;}\left(h\;V_{m}\;V_{m}\right)=\frac{-h\nabla_{s\;}p_{m}}{\rho_{m}}+g_{\tau }h+\frac{3}{2\rho_{m}}\tau_{fs}-\frac{3v}{h}V_{m}+\frac{q}{\rho_{m}}$$ (4)

where *p*_*m*_ represents the pressure within the mucus film, *g*_*τ*_ represents the effect of gravity in the direction parallel to the film, *τ*_*fs*_ corresponds to the shear stress acting on the film surface, *ν* is the kinematic viscosity, *q* refers to the momentum source term.

The onset of mucus film breakup is modeled using a Weber number-based stripping criterion, which quantifies the balance between inertial forces from the airflow and the surface tension of the mucus film. In this study, a critical Weber number of 0.03 was selected to initiate film stripping, based on calibration with experimental data on droplet size and number during the cough event. This low threshold reflects the thin mucus film (20–30 µm) and high airflow velocities in the airway, which amplify inertial forces relative to surface tension, promoting rapid film breakup into ligaments and droplets.

The Discrete Phase Model (DPM) was used to track the motion of droplets in the airway. The DPM uses the Euler-Lagrange approach, where the Navier-Stokes equations describe the fluid flow, and Newton’s laws of motion govern the movement of discrete particles, such as droplets, within the flow. The dispersed phase interacts with the continuous fluid phase by exchanging momentum, mass, and energy. This interaction influences the movement of each droplet within the fluid flow. The position of droplets is determined based on their interactions with the fluid and the forces acting on them. The droplets trajectory is predicted by integrating the forces acting on them. The model considers various forces, including viscous drag and gravity, which are crucial in determining the trajectory, dispersion, and overall behavior of the droplets. Additionally, it accounts for the stochastic behavior of the surrounding turbulent flow.

Droplet impingement on the airway walls was modeled using the Stanton-Rutland model, which determines whether droplets stick, rebound, or splash based on impact parameters such as the Weber number and the Reynolds number. This model governs the interaction between droplets and the mucus film, influencing reabsorption outcomes. For simplicity, the contact angle of the mucus film was not considered in this study, assuming uniform droplet-wall interactions.

Cough airflow exhibits inherently turbulent behavior due to the high Reynolds number and complex geometry of the upper airway. The Shear Stress Transport (SST) k–ω model, a Reynolds-Averaged Navier-Stokes (RANS) approach, was employed to model this turbulence. The model solves two transport equations: one for turbulent kinetic energy (k) and another for the specific dissipation rate (ω), from which the turbulent viscosity is derived, effectively capturing turbulence effects without resolving all flow scales.

### 2.3 Boundary conditions

**FIGURE 2:**
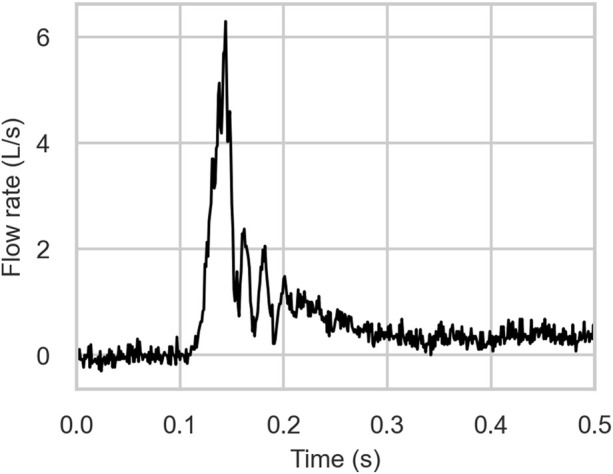
Transient cough waveform profile.

The cough airflow was modeled as an isothermal, incompressible ideal gas with a density of 1.225 kg/m^3^ and a dynamic viscosity of 1.78 × 10^−5^ Pa·s. A transient cough flow was simulated using an experimentally derived cough profile as the inlet boundary condition (Figure 2). The time-resolved voluntary cough waveform was acquired using an oral pneumotachograph coupled with a spirometer, following the protocol described in (Ilegbusi et al., 2021). The cough flow was implemented through a user-defined function (UDF) in ANSYS Fluent to define the time-varying mass flow rate at the distal ends of the seven bronchi. A zero-gauge pressure condition was applied at the mouth opening, assuming atmospheric pressure during the cough. All airway walls were subjected to a no-slip condition, and the effects of gravity was included in the simulation.

To ensure mass conservation in the computational model, the total cough airflow rate, derived from the experimentally obtained cough profile, was uniformly divided among the seven inlet boundaries located at the distal ends of the segmental bronchi. This uniform distribution assumes equal mass flow across each inlet, simplifying the representation of bronchial flow partitioning in the absence of specific experimental data on regional flow variations.

### 2.4 Computational details

Simulations were conducted using ANSYS Fluent 2023 R1, employing a transient pressure-based solver. A time step of 0.0001 s was used for the simulation. Residual convergence criteria were set to 10^–4^ for continuity, momentum, and turbulence equations. All simulations were performed on a high-performance computing (HPC) cluster using a single computational node with 16 threads on an Intel Xeon 64-bit processor. The total computation time per simulation was approximately 48 h.

### 2.5 Pathological cases considered

This study investigates the impact of mucus thickness and viscosity on cough-induced droplet dynamics in the upper airway, representing a spectrum of respiratory conditions from healthy to diseased states. Three mucus thicknesses (20 μm, 25 μm, and 30 µm) and three values of viscosity (0.001003 kg/m·s, 0.001254 kg/m·s, and 0.001505 kg/m·s) were used to parametrically model progressive mucus thickening and increased viscosity—hallmarks of airway diseases. These parameters capture physiological variations from normal to pathological states. Table 1 summarizes the simulated cases.

**TABLE 1:** Simulated Cases for Mucus Thickness and Viscosity representing Healthy and Pathological Airway Conditions.

Case	Thickness (µm)	Viscosity (kg/m·s)	Description
Type I	20	0.001003	Healthy baseline
Type II-A	25	0.001003	Moderate thickening, baseline viscosity
Type II-B	25	0.001254	Moderate thickening, moderate viscosity
Type II-C	25	0.001505	Moderate thickening, high viscosity
Type III	30	0.001003	Advanced thickening, baseline viscosity

#### 2.5.1 Type I: healthy baseline

The Type I case (20 μm, 0.001003 kg/m·s) represents a healthy human airway. The thickness aligns with reported values of 10–20 µm for healthy mucus layers (Atanasova and Reznikov, 2019; Duncan et al., 2016; Michael Foster, 2002), and the viscosity corresponds to water-like mucus, enabling effective mucociliary clearance without obstructing airflow (Button and Button, 2013). This case serves as the reference baseline for comparison.

#### 2.5.2 Type II and Type III: pathological thickness and viscosity variations

Direct *in vivo* measurements of mucus thickness in respiratory diseases such as COPD, cystic fibrosis, and chronic bronchitis are limited due to patient variability and imaging challenges. However, the literature reports significant mucus thickening and increased viscosity resulting from hypersecretion, dehydration, or airway inflammation (Abrami et al., 2024; Fahy and Dickey, 2010; Hill et al., 2022). To model these pathological changes, Type II (25 µm) and Type III (30 µm) were investigated, representing moderate (25% increase) and advanced (50% increase) mucus thickening relative to the healthy baseline.

To investigate the role of viscosity in droplet dynamics, three viscosity values were simulated for the Type II thickness (25 µm): Type II-A (0.001003 kg/m·s, baseline), Type II-B (0.001254 kg/m·s, 25% increase), and Type II-C (0.001505 kg/m·s, 50% increase). These values reflect moderate to advanced pathological viscosity changes while remaining physiologically plausible. Type II was selected for viscosity variations due to its intermediate thickness, which is characteristic of early-to-moderate disease progression.

These incremental changes in thickness and viscosity ensure that the simulations capture physiologically relevant variations in the upper airway mucus layer, enhancing the clinical relevance of the study.

## 3 Results

### 3.1 Validation

In our previous work (Khan and Ilegbusi, 2025), we validated the cough airflow in a patient-specific airway model by comparing the simulation results with experimental data from Rochefort et al., who employed magnetic resonance phase-contrast velocimetry technique to measure the three-dimensional velocity components in a 3D airway model (De Rochefort et al., 2007). The computed results were compared with experimental measurements at the midsection of the right main bronchus, focusing on two velocity profiles along orthogonal lateral directions: Left-Right (LR) and Anterior-Posterior (AP). The comparison showed agreement at boundary locations; however, discrepancies within the domain were attributed to differences in the 3D geometries between the studies. To quantify errors, the Mean Absolute Error (MAE) and Mean Squared Error (MSE) were calculated for both profiles. For the AP profile, the MAE was 0.5056, and the MSE was 0.3050, suggesting moderate discrepancies between the experimental and CFD velocities. For the LR profile, the MAE was 0.3312, and the MSE was 0.189, indicating better agreement between the CFD and experimental measurements. These errors highlight areas for potential refinement in the CFD model, but the model captured the general trends in the flow behavior within the airway.

Additionally, a comparison with the numerical results of (Ren et al., 2022) revealed similar trends in velocity profiles at the first bifurcation of the right main bronchus. The errors between our results and those of Ren’s were also quantified. For the AP profile, the MAE was 0.1199, and the MSE was 0.0308. For the LR profile, the MAE was 0.1188, and the MSE was 0.0251, demonstrating strong agreement between the two models.

The exhaled droplet size distribution from the CFD results was compared with experimental data from (Xie et al., 2009). Both datasets exhibit a right-skewed distribution with a dominant size range of 50–100 μm, accounting for 54.2% (experimental) and 59.5% (CFD) of total droplets. The CFD model predicts a mean droplet size of 90.4 µm, closely aligning with the experimental mean of 86.2 µm, and both distributions show no droplets below 24 μm, consistent with measurements at the mouth exit. The total exhaled droplet count from the CFD model (791 droplets) is in close agreement with the experimental average of 800 droplets from four healthy subjects, with a relative difference of ∼1.1%.

These minor discrepancies may arise from assumptions in the CFD model, which do not fully account for inter-subject variability or non-Newtonian mucus behaviour observed *in vivo*. Nevertheless, the strong agreement in total droplet count, mean size, and dominant size range validates the CFD model’s ability to accurately reproduce the biomechanical processes governing cough-induced droplet formation.

### 3.2 Mechanics of droplet formation

**FIGURE 3:**
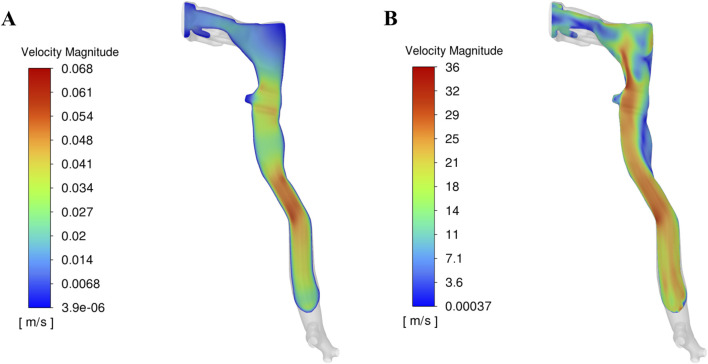
Velocity profile in the upper airway at two time durations: **(A)** Before onset of peak cough at 0.1 s, **(B)** During peak cough flow at 0.15 s.

The velocity profiles of the cough flow at two different time durations are presented in Figure 3. The color map represents velocity magnitude, with red regions indicating high velocity and blue regions indicating low velocity. Air flows from the terminal bronchioles, converges in the trachea, and exits through the mouth. As the air passes from the peripheral to the central airways, the velocity increases due to the decreasing cross-sectional area. After 0.1 s, the expiratory phase begins (see Figure 2), driven by high intrathoracic pressures from the preceding compressive phase. This results in a brief, intense burst of turbulent airflow, with peak velocity occurring at approximately 0.15 s and reaching more than 36 m/s (Figure 3). This peak cough flow results from the forceful expulsion of air from the compressed central airways.

**FIGURE 4:**
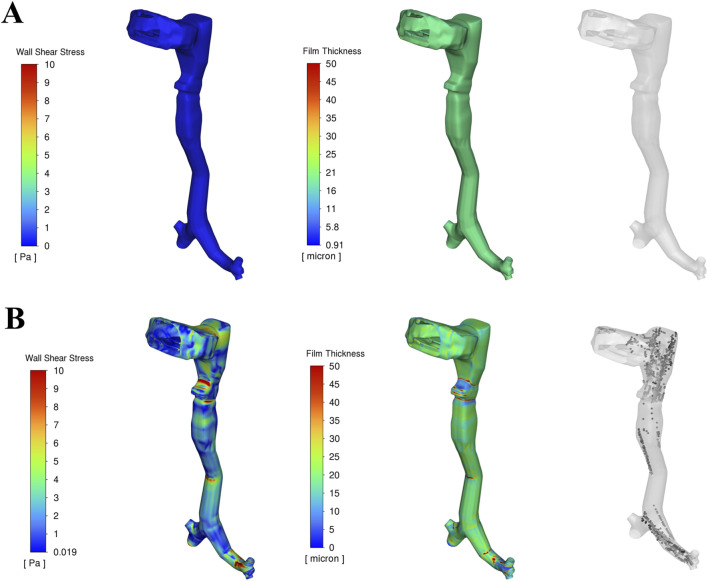
Wall shear stress, Mucus film dynamics and droplet generation in the upper airway at two time durations: **(A)** Before onset of peak cough at 0.1 s, **(B)** During peak cough flow at 0.15 s.

Figure 4 presents the corresponding wall shear stress, mucus film, and droplet distributions at the two time points analysed in Figure 3. The red regions indicate high shear stress or thick mucus, while blue regions represent low values. Before the onset of expiratory flow at 0.1 s, airflow velocity is low, resulting in minimal shear stress on the airway walls. Consequently, the mucus film remains intact, and no droplets are generated. The uniform 20 µm mucus layer shows no significant thinning or detachment at this stage (Figure 4A).

As the expiratory phase progresses, airflow velocity increases rapidly. During peak cough flow at 0.15 s, high-velocity air generates increased shear stress—up to approximately 15 Pa—particularly in the laryngeal region and terminal airways, where geometric constrictions amplify local velocities. This leads to disruption of the mucus film, with the film thickness reducing to nearly 0 µm in high-shear regions. This process results in aerosolization, generating mucus-derived droplets from the airway surface (Figure 4B).

These areas of maximum shear align closely with zones of intense airflow acceleration. In contrast, regions exposed to lower shear stress retain much of their initial film thickness, resulting in spatial variability in droplet generation. The findings emphasize that shear stress magnitude—driven by airflow velocity—is the dominant factor influencing mucus detachment and droplet formation during a cough.

### 3.3 Droplet fate

During the cough event, droplets generated from the mucus film follow one of three possible trajectories: exhalation from the airway, redeposition into the mucus layer, or retention within the airway. The fate of these droplets is governed by a complex interplay of physical forces, including droplet inertia, viscous drag, gravity, turbulent dispersion, surface adhesion, and interactions with the mucus film. During peak cough flow (∼36 m/s, Figure 3), high shear stress (up to 15 Pa, Figure 4) induces instabilities in the mucus film, leading to breakup and droplet formation. The likelihood of a droplet being exhaled or reabsorbed depends on its size, velocity, and interactions with the airway’s complex geometry and airflow dynamics.

Droplet inertia, governed by Newton’s laws, plays a critical role in determining their trajectory. Larger droplets (>100 µm) possess higher inertia, enabling them to overcome drag and gravitational forces and exit the airway through the oral cavity, resulting in exhalation. Smaller droplets (<50 µm), however, are more susceptible to turbulent dispersion, increasing their likelihood of redeposition onto the airway walls. Geometrically complex regions such as the larynx and bronchial bifurcations create local eddies and recirculation zones that trap smaller droplets, promoting redeposition. Surface adhesion further influences redeposition: the mucus film’s surface tension determines the energy required for droplet detachment, while droplets in lower-shear regions reattach due to adhesive forces, reintegrating into the mucus layer.

A total of 14,033 droplets is generated during the high-velocity expiratory phase. Of these, 791 droplets (∼5.6%) are exhaled from the airway, primarily mid-sized droplets (50–125 µm) carried by the high-velocity airflow, while the remaining 9,395 droplets (∼94.4%) are redeposited into the airway mucus film, driven by gravitational settling, turbulent dispersion, and adhesion in lower-velocity regions. No droplets are retained in the airway at the end of the simulation, indicating efficient clearance through either exhalation or absorption. These findings highlight the importance of airflow dynamics and mucus properties in determining droplet trajectories, with implications for pathogen transmission and airway clearance mechanisms.

### 3.4 Effect of mucus thickness and viscosity on exhaled and absorbed droplet size distribution

This section presents the effects of pathological changes in mucus thickness and viscosity (and consequently, the disease state) on droplet generation and size distribution during a cough. Simulations were performed across a range of mucus thicknesses and viscosities. Three mucus thicknesses were considered: Type I (20 µm), Type II (25 µm), and Type III (30 µm). Additionally, three viscosity values were considered: Type II-A (0.001003 kg/m·s), Type II-B (0.001254 kg/m·s), and Type II-C (0.001505 kg/m·s). These variations, reflecting pathological changes from the baseline case, significantly influence the size distribution of exhaled and absorbed droplets during a cough.

#### 3.4.1 Exhaled droplets

As mucus thickness increases (correspondingly, disease severity), the total number of exhaled droplets rises with a broader size distribution. For Type I mucus (healthy baseline), exhaled droplets are predominantly mid-sized (50–125 µm), peaking at 75–100 µm. In contrast, Type III mucus (advanced thickening) generates a higher droplet count, with a notable increase in the mean size of droplets. No droplets smaller than approximately 25 µm were observed across any mucus thickness conditions. Summary statistics are presented in Table 2. Results indicate that thicker mucus, associated with disease conditions, produces larger and more varied droplet sizes.

**TABLE 2:** Summary statistics of exhaled and absorbed droplet size distributions for varying mucus thickness.

Droplet fate	Mucus thickness	Count	Min (µm)	Max (µm)	Mean (µm)	Std Dev (µm)
Exhaled droplets	Type I	791	27.9	409.4	94.64	49.24
	Type II	1700	24.93	741.14	105.82	55.56
	Type III	3,446	25.17	587.44	114.82	60.52
Absorbed droplets	Type I	16,123	10.02	1,131.8	105.44	63.99
	Type II	37,855	10.30	1,358.20	133.69	90.76
	Type III	84,723	10.26	1985.4	169.66	139.79

As mucus viscosity increases (and correspondingly, disease severity), the total number of exhaled droplets decreases, but the droplet size distribution shifts toward larger sizes with increased variability. Type II-A (baseline viscosity) produces most droplets sized (50–150 µm), peaking at 75–100 µm. Type II-B (moderate viscosity) and Type II-C (high viscosity) produce fewer droplets with larger mean sizes and broader distributions. No exhaled droplets smaller than approximately 25 µm are observed for Type II-A, with higher minimum sizes for Type II-B and Type II-C. Summary statistics are presented in Table 3. Results suggest that higher mucus viscosity, associated with disease severity, produces fewer exhaled droplets but yields larger droplets with a broader size distribution.

**TABLE 3:** Summary statistics of exhaled and absorbed droplet size distributions for varying mucus viscosities.

Droplet fate	Mucus viscosity	Count	Min (µm)	Max (µm)	Mean (µm)	Std Dev (µm)
Exhaled droplets	Type II-A	1700	24.93	741.14	105.82	55.56
	Type II-B	1,047	38.99	534.76	110.91	52.45
	Type II-C	624	39.60	571.38	115.27	57.74
Absorbed droplets	Type II-A	37,855	10.30	1,358.20	133.69	90.76
	Type II-B	18,482	12.13	1,531.50	122.84	77.41
	Type II-C	12,601	13.80	1,032.30	118.36	69.87

#### 3.4.2 Absorbed droplets

Absorbed droplets—those redepositing into the mucus layer—follow a similar trend to exhaled droplets. Thicker mucus produces more absorbed droplets and larger mean sizes, with a notable proportion of droplets exceeding 200 µm in Type III. No absorption occurs for droplets below 10 µm (Table 2). Compared to exhaled droplets, absorbed droplets are consistently greater in number and exhibit larger mean sizes across all mucus conditions. Thus, increasing mucus thickness consistently results in higher droplet counts and larger droplet sizes for both exhaled and absorbed droplets.

Higher viscosity (Type II-B and Type II-C) results in fewer absorbed droplets compared to Type II-A (baseline), as the number of droplets generated is reduced. The mean size of absorbed droplets decreases slightly, reflecting a trend toward smaller droplet sizes with increasing viscosity. No absorption occurs for droplets smaller than 10 µm across all conditions (Table 3). Compared to exhaled droplets, absorbed droplets are consistently greater in number but exhibit slightly smaller mean sizes at higher viscosities, with a narrower size distribution. These results suggest that increased viscosity enhances absorption efficiency while favoring slightly smaller droplet sizes.

**FIGURE 5:**
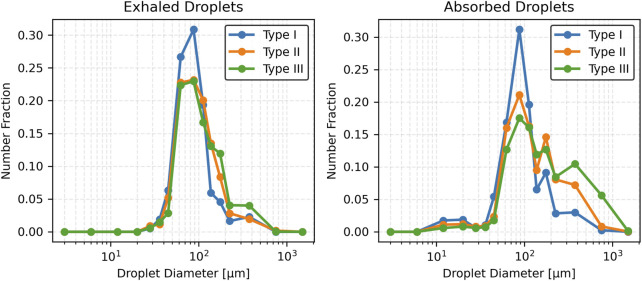
Number fraction of droplets for varying mucus thicknesses.

Figure 5 shows the number fraction of exhaled and absorbed droplets across size ranges. The Type I mucus (healthy condition) yields the highest peak number fraction, particularly in the 75–100 µm range. Conversely, the Type II and Type III (disease conditions) show broader distributions with reduced peaks and a greater prevalence of larger droplets (>125 µm). Notably, droplets exceeding 200 µm become prominent in the Type III (advanced thickening) case. This reinforces the observed trend that thicker mucus (more severe disease) shifts the distribution toward larger droplet sizes, while thinner mucus (less severe disease) concentrates droplets in a narrower mid-size range.

**FIGURE 6:**
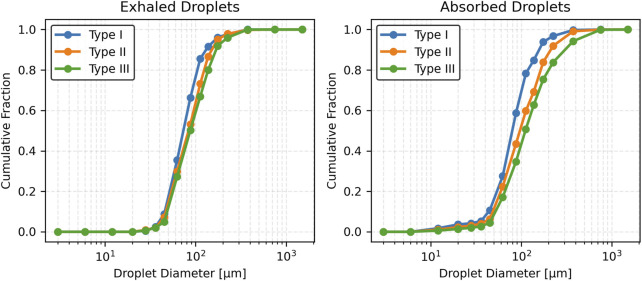
Cumulative fraction of droplets for varying mucus thicknesses.

Figure 6 presents cumulative distributions for both exhaled and absorbed droplets. The curve for Type I mucus (healthy condition) rises steeply and plateaus early, reflecting the dominance of smaller droplets (<100 µm). In contrast, Type II and Type III (disease conditions) curves rise more gradually and extend to larger sizes, particularly for Type III, which skews heavily toward >125 µm. Absorbed droplet distributions follow similar trajectories, with thicker mucus associated with a slower, broader rise—indicating retention of a higher proportion of large droplets.

**FIGURE 7:**
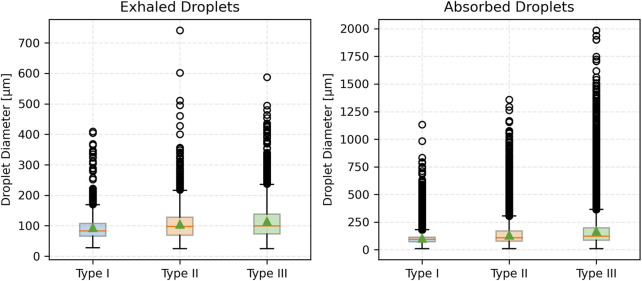
Box plot of droplet diameter distributions across varying mucus thicknesses.

Figure 7 compares droplet diameter distributions through box plots. The median size of exhaled droplets increases with mucus thickness but remains consistently lower than that of absorbed droplets. Absorbed droplets exhibit greater variability, with wider interquartile ranges and more outliers, especially for Type III (most severe disease) mucus thickness. In contrast, exhaled droplets from thinner mucus (less severe disease) show a narrower size spread, underscoring the uniformity of smaller droplet formation.

**FIGURE 8:**
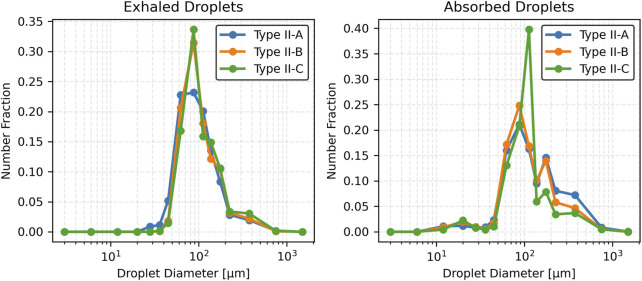
Number fraction of droplets across varying mucus viscosities.

Figure 8 illustrates the number fraction of exhaled and absorbed droplets for the three viscosity values considered. For Type II-A (baseline viscosity), the distribution peaks in the 75–100 µm range, indicating a concentration of mid-sized droplets. In comparison, Type II-B (moderate viscosity) and Type II-C (high viscosity) show slightly broader distributions, also peaking in the 75–100 µm range. Notably, Type II-C produces a greater proportion of larger droplets (>125 µm) than the baseline. Droplets exceeding 500 µm remain rare across all conditions. For absorbed droplets, Type II-A also peaks at 75–100 µm but exhibits a notable presence of larger droplets in the 150–200 µm range. Type II-B and Type II-C follow similar patterns, with peak probabilities also in the 75–100 µm range. However, Type II-C shows an increased probability in the 100–125 µm range, indicating a viscosity-dependent shift toward larger absorbed droplets. Larger droplets (>250 µm) are more prominent in the absorbed droplet distributions, especially for Type II-A.

**FIGURE 9:**
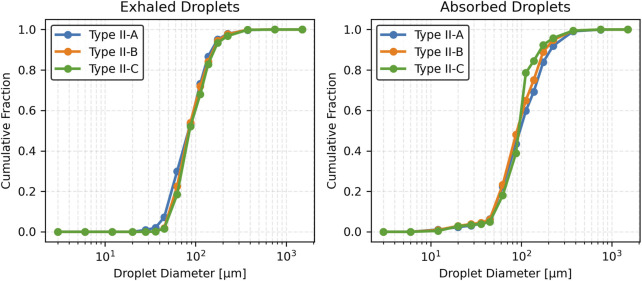
Cumulative fraction of droplets across varying mucus viscosities.

Figure 9 presents the cumulative distribution functions (CDFs) of exhaled and absorbed droplet sizes. For exhaled droplets, the CDF for Type II-A rises sharply and plateaus early, reflecting a predominance of smaller droplets (<100 µm). In contrast, Type II-B and Type II-C exhibit more gradual increases, with the curve for Type II-C extending further toward larger droplet sizes (>125 µm), consistent with its higher mean and standard deviation. For absorbed droplets, Type II-A shows a more gradual cumulative increase, with a significant contribution from droplets >125 µm. Type II-B and Type II-C rise even more slowly, with Type II-C displaying a pronounced skew toward larger sizes (100–125 µm), indicating increased retention of larger droplets as viscosity rises.

**FIGURE 10:**
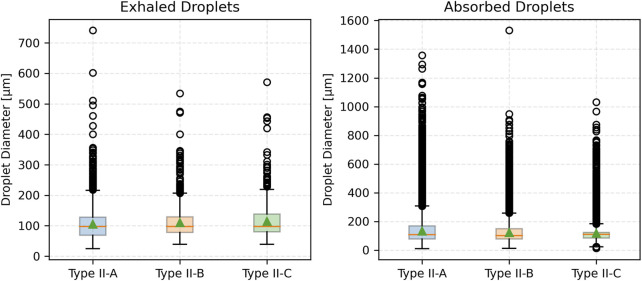
Box plot of droplet diameter distributions across varying mucus viscosities.

Figure 10 displays box plots of droplet diameters for both exhaled and absorbed droplets. For exhaled droplets, the median size increases slightly with viscosity from Type II-A to Type II-C, with greater variability. In contrast, absorbed droplets consistently have larger median sizes, increasing from Type II-A to Type II-C. These droplets exhibit larger variability, with wider interquartile ranges and more outliers, particularly for Type II-A. Exhaled droplets under lower viscosity maintain narrower size distributions, reflecting uniform formation of smaller droplets in those cases.

**FIGURE 11:**
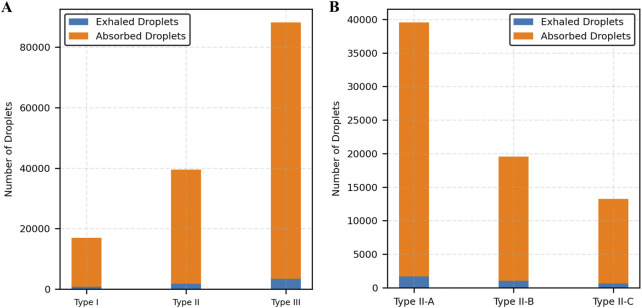
Distribution of exhaled and absorbed droplets. **(A)** Varying mucus thickness, **(B)** Varying mucus viscosity.

Figure 11a illustrates the partitioning of droplet counts between exhaled and absorbed droplets. Across all mucus thicknesses, absorbed droplets account for over 90% of total droplet generation. The proportion of absorbed droplets increases with thicker mucus, demonstrating the enhanced absorption capability of thicker mucus layers. Figure 11b illustrates the partitioning of droplet counts between exhaled and absorbed droplets for a fixed mucus thickness (25 µm) across varying viscosity levels. Absorbed droplets consistently account for over 95% of total droplet generation in all cases. As viscosity increases (Type II-A to Type II-C), the proportion of absorbed droplets slightly increases, while the total number of droplets decreases, indicating that higher viscosity enhances absorption efficiency but reduces overall droplet production.

### 3.5 Statistical analysis

Statistical analysis was conducted for exhaled droplet size distributions given their direct relevance to clinical measurements. Absorbed droplets, while showing similar qualitative trends in size distribution, were excluded from statistical analysis because they remain within the airway and are not measurable non-invasively, limiting their applicability to external diagnostic methods.

The Kruskal-Wallis test was applied to assess differences in exhaled droplet size distributions across cases with varying mucus thickness (Type I, Type II, and Type III) and viscosity levels (Type II-A, Type II-B, Type II-C). This non-parametric test, implemented using the kruskal function from SciPy (version 1.10.1) in Python (version 3.9), was selected due to the non-normal, right-skewed nature of the data. The results confirmed significant effects of both mucus thickness (H = 105.60, p < 0.0001) and viscosity (H = 20.21, p < 0.0001) on droplet size distributions.

Post-hoc pairwise comparisons were conducted using the Mann-Whitney U test with a Bonferroni correction (α = 0.05/3 = 0.0167) to account for multiple comparisons across the three pairs of mucus thickness (Type I vs. Type II, Type II vs. Type III, Type I vs. Type III) and viscosity (Type II-A vs. Type II-B, Type II-A vs. Type II-C, Type II-B vs. Type II-C). The tests were performed using the mannwhitneyu function from SciPy with the alternative = 'two-sided’ parameter. For mucus thickness, significant differences were observed in all pairs: Type I vs. Type II (U = 568,446, p < 0.0001), Type II vs. Type III (U = 1,055,384, p < 0.0001), and Type I vs. Type III (U = 2,692,529, p < 0.0001). For mucus viscosity, significant differences were found in two pairs: Type II-A vs. Type II-B (U = 824,740, p < 0.01) and Type II-A vs. Type II-C (U = 472,846, p < 0.01), but no significant difference was observed between Type II-B and Type II-C (U = 316,186, p > 0.01).

Droplet size data were extracted directly from the Discrete Phase Model outputs in ANSYS Fluent 2023 R1 without additional preprocessing and analyzed using Python (version 3.9) with SciPy (version 1.10.1). These findings quantitatively support the observation that increased mucus thickness and viscosity (and correspondingly, disease severity) significantly influences exhaled droplet size distributions, highlighting their direct impact on both the number and size of droplets generated during a cough.

## 4 Discussion

This study presents a computational framework that simulates droplet generation and transport during a cough event, incorporating realistic airway geometry and mucus film behavior for the classification of pulmonary disease. By integrating the Eulerian Wall Film model and Discrete Phase Model within a validated CFD environment, the framework provides a mechanistic understanding of how variations in mucus thickness and viscosity, representative of healthy and pathological airway conditions, influence droplet formation and fate.

Mucus thickness and viscosity significantly influence droplet dynamics, with implications for respiratory pathophysiology, such as in COPD or cystic fibrosis. The results demonstrate that increasing mucus thickness from healthy (Type I) to intermediate (Type II) and advanced (Type III) pathological states amplifies shear-induced droplet generation, shifting exhaled droplet size distributions toward larger sizes and higher counts (Table 2; Figures 5–7). Similarly, increasing mucus viscosity from baseline (Type II-A) to moderate (Type II-B) and high (Type II-C) conditions shifts exhaled droplet size distributions toward larger sizes (>125 µm), with higher variability and increased median droplet sizes. In contrast, lower viscosity conditions (Type II-A) produce smaller, more uniform droplets (<100 µm) (Table 3; Figures 8–10). Higher viscosity increases resistance to airflow-induced shear, promoting the formation and retention of larger droplets. These viscosity- and thickness-driven changes in droplet dynamics are critical for understanding disease progression and developing non-invasive diagnostic biomarkers based on exhaled droplet characteristics.

The observed shifts in droplet size and count have significant implications for disease progression and pathogen transmission. Larger droplets, more common in intermediate (Type II) and advanced (Type III) pathological conditions, may exacerbate airway obstruction by increasing mucus retention and pathogen load in diseased airways. Larger droplets also increase the short-range transmission risk of respiratory pathogens like SARS-CoV-2, in close-contact settings, such as healthcare facilities or poorly ventilated indoor spaces. This transmission risk underscores the need for targeted infection control measures, including high-efficiency masks and optimized ventilation systems. These findings can inform pathogen-specific transmission models and guide public health strategies, such as designing masks tailored for patients with respiratory diseases and developing ventilation systems to mitigate risks from larger droplets.

In addition to exhaled droplets, which serve as potential non-invasive diagnostic biomarkers, the study analyzed absorbed droplets—those redepositing into the mucus layer—to provide a comprehensive view of droplet dynamics during a cough. Absorbed droplets, constituting over 90% of generated droplets across all mucus thicknesses and viscosities, contribute to airway clearance by reintegrating into the mucus layer. This process may influence pathogen retention and airway obstruction in diseased states. While absorbed droplets have limited diagnostic utility due to their inaccessibility for non-invasive measurement, their analysis enhances understanding of mucus-driven droplet mechanics, informing models of airway clearance efficiency and pathogen transmission risks.

Although the present model captures key biomechanical interactions, it has limitations that impact its scope. The mucus layer was modeled as a Newtonian fluid, whereas real mucus exhibits shear-thinning properties. The model assumes rigid airway walls, which do not account for airway compliance observed in real respiratory systems, particularly during coughs. These assumptions were made to reduce computational complexity and focus on mucus properties as the primary variable, as it plays a dominant role in droplet generation.

Future work could validate these models against *in vitro* airway models or clinical droplet measurements to enhance translational impact. Incorporating non-Newtonian rheology, such as Power-Law or Carreau-Yasuda models, and compliant airway walls could enhance the model’s fidelity in capturing mucus-droplet interactions.

In summary, this study provides a physics-based foundation for understanding how disease-driven changes in mucus properties influence droplet generation and transport. The findings support non-invasive diagnostics via exhaled droplet characteristics, personalized therapeutic interventions, and targeted infection control measures for respiratory diseases.

## Data Availability

The raw data supporting the conclusions of this article will be made available by the authors, without undue reservation.
